# LeTra: a leaf tracking workflow based on convolutional neural networks and intersection over union

**DOI:** 10.1186/s13007-024-01138-x

**Published:** 2024-01-17

**Authors:** Federico Jurado-Ruiz, Thu-Phuong Nguyen, Joseph Peller, María José Aranzana, Gerrit Polder, Mark G. M. Aarts

**Affiliations:** 1 Center for Research in Agricultural Genomics (CRAG), Cerdanyola, 08193 Barcelona, Spain; 2 Laboratory of Genetics, Wageningen University and Research (WUR), Droevendaalsesteeg 1, 6708 PB Wageningen, The Netherlands; 3 Greenhouse Horticulture, Wageningen University and Research (WUR), Wageningen, The Netherlands; 4 Institut de Recerca i Tecnologia Agroalimentàries (IRTA), Barcelona, Spain

**Keywords:** Phenotyping, Tracking, Photosynthesis, Convolutional neural networks, Arabidopsis, Image analysis

## Abstract

**Background:**

The study of plant photosynthesis is essential for productivity and yield. Thanks to the development of high-throughput phenotyping (HTP) facilities, based on chlorophyll fluorescence imaging, photosynthetic traits can be measured in a reliable, reproducible and efficient manner. In most state-of-the-art HTP platforms, these traits are automatedly analyzed at individual plant level, but information at leaf level is often restricted by the use of manual annotation. Automated leaf tracking over time is therefore highly desired. Methods for tracking individual leaves are still uncommon, convoluted, or require large datasets. Hence, applications and libraries with different techniques are required. New phenotyping platforms are initiated now more frequently than ever; however, the application of advanced computer vision techniques, such as convolutional neural networks, is still growing at a slow pace. Here, we provide a method for leaf segmentation and tracking through the fine-tuning of Mask R-CNN and intersection over union as a solution for leaf tracking on top-down images of plants. We also provide datasets and code for training and testing on both detection and tracking of individual leaves, aiming to stimulate the community to expand the current methodologies on this topic.

**Results:**

We tested the results for detection and segmentation on 523 *Arabidopsis thaliana *leaves at three different stages of development from which we obtained a mean F-score of 0.956 on detection and 0.844 on segmentation overlap through the intersection over union (IoU). On the tracking side, we tested nine different plants with 191 leaves. A total of 161 leaves were tracked without issues, accounting to a total of 84.29% correct tracking, and a Higher Order Tracking Accuracy (HOTA) of 0.846. In our case study, leaf age and leaf order influenced photosynthetic capacity and photosynthetic response to light treatments. Leaf-dependent photosynthesis varies according to the genetic background.

**Conclusion:**

The method provided is robust for leaf tracking on top-down images. Although one of the strong components of the method is the low requirement in training data to achieve a good base result (based on fine-tuning), most of the tracking issues found could be solved by expanding the training dataset for the Mask R-CNN model.

## Background

There are increasingly more high throughput (HTP) phenotyping technologies becoming available, which provides increasingly more methods to support plant breeding efforts for crop improvement. HTP phenotyping platforms are largely based on a wide range of imaging technologies, including visible light (blue 450 nm, green 550 nm and red 600 nm), chlorophyll fluorescence (CF), thermal, hyperspectral imaging and more, reviewed in [1]. As a result, a large number of plants at various developmental stages can be measured automatically in a nondestructive manner for target phenotypes. Such large-scale screening enables the selection of the best performing genotypes as well as best phenotypes as predictors of complex traits such as stress tolerance, disease resistance and yield. At the same time the genetic basis of complex traits can be revealed in genetic studies where high-density genetic makers and accurate phenotypes are associated with large-scale population (genetic mapping for quantitative trait locus). Photosynthesis is considered a difficult trait for breeding or for genetic studies because of its polygenic nature and high sensitivity to environmental changes. This makes photosynthesis parameters a good indicator of biotic and abiotic stresses as well as plant productivity that potentially associates with yield [2–4]. CF imaging is becoming a powerful and popular technique for measuring efficiency of photosystem II (ϕPSII). The HTP phenotyping platform using CF imaging, the so-called Phenovator [5], was developed at Wageningen University, which led to successful studies on natural variation for photosynthesis in the model species *Arabidopsis thaliana* [6, 7]. An advanced photosynthesis phenotyping platform, built by Photon Systems Instruments (PSI), is now embedded in the Netherlands Plant Eco-phenotyping Center (NPEC; www.npec.nl), which setup is now available for *A. thaliana* and other species with various plant sizes and architectures. The current image analysis does not include automated individual leaf tracking over time, but as phenotypic variation in leaf morphology and other, e.g. photosynthesis, parameters are observed between developmental stages and between leaves [8–10], the development of leaf detection and segmentation methods on images is required. Many methods have been developed for leaf segmentation [11–13], but recent advances in artificial intelligence (AI) allow for new approaches. The implementation of the most recent tools for segmentation, such as Mask-RCNN [14] remains to be explored. These models have the advantage of performing both detection and segmentation in one step and being fast in inference times, simplifying the pipeline. All these methods (AI based or not) still have a problem on time series association. The instance detection order varies, depending on the position of the leaves, requiring an association step to produce a time series for each individual leaf which might be challenging to implement [15]. As an alternative, we find the use of complex AI methods capable of both tasks [16, 17], but these methods require abundant training data, which might be expensive to produce timewise. Therefore, in this manuscript we suggest a hybrid approach combining convolutional neural networks (CNN) to solve detection and segmentation, with a numerical method to perform association between time points. We also present an analysis of photosynthesis data of two Arabidopsis accessions (Columbia-0 and Ely) as a case study. Results showed that the photosynthetic capacity and photosynthetic response to light treatments are influenced by leaf age and order. There is variation in leaf-dependent photosynthesis, which is of great interest for quantitative genetic studies. The main contributions of this research are summarized as follows: (1) A study on the efficiency of Mask R-CNN to individualize leaves in Arabidopsis. (2) A method based on intersection over union (IoU) to track individual leaf growth on temporal series. (3) A novel comparative study of the photosynthesis efficiency between Columbia-0 and Ely at individual leaf level. (4) A repository with code and a dataset to recreate the work and train other detection models.

## Results

### General approach

**Fig. 1:**
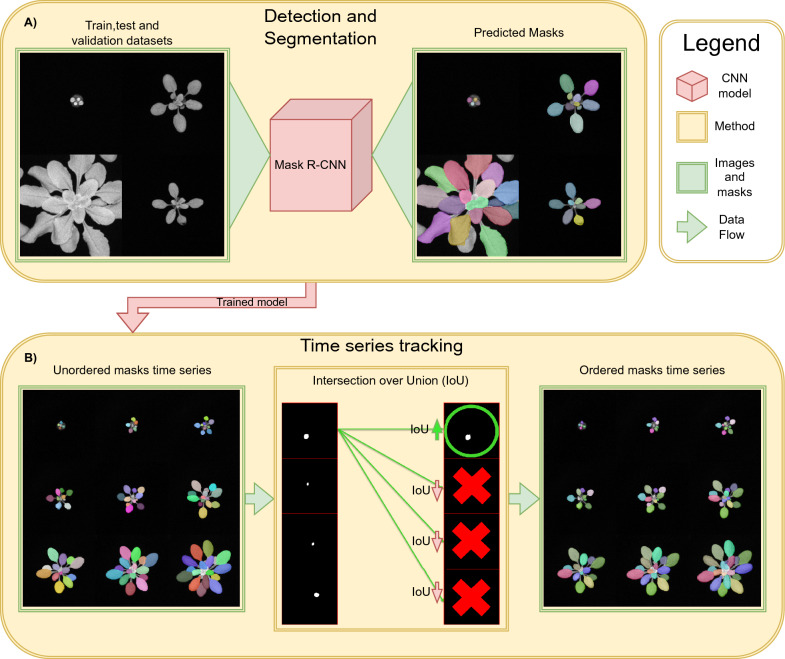
Graphical abstract of the proposed approach, consisting of two main stages. In the first stage (**A**), a detection and segmentation model is trained using images of *A. thaliana* rosettes. In the second stage (**B**), the trained model is employed to detect and segment individual images at each time step. The resulting masks are ranked according to their IoU as denoted by green and red arrows representing high and low IoU values, respectively. This ranking enables the association and reconstruction of object tracking

The proposed method first employs Mask R-CNN to detect leaves and generate their corresponding binary masks (Fig. 1A). Then, the IoU is used to measure the similarity between the masks in consecutive frames and find the correspondence between them (Fig. 1B). This correspondence information is further utilized to track the leaves over time. The experimental results demonstrated the effectiveness of the proposed method in accurately detecting and tracking leaves in a top-down setting.

### Leaf detection and segmentation

A total of 523 different leaves were evaluated, categorized by origin. A total of 304 came from plants in the last stages of development, 151 at the mid stages and 68 at the very early stages. In the first contrast we evaluated how many of them were effectively detected (True positives, TP) against how many of them were not (False negatives, FN) and against how many false detections (False positives, FP) were found. From these leaves we found 294/10/26 (TP/FN/FP) for the late stage, 146/5/7 (TP/FN/FP) for the mid stage and 65/3/1 (TP/FN/FP) for the early stages. Based on these values we estimated the precision (P), recall (R) and F-score (Fs) for each stage, having 0.9187/0.9671/0.9423 (P/R/Fs) for the late stage, 0.954/0.969/0.96 (P/R/Fs) for the mid stage and 0.985/0.9558/0.9701 (P/R/Fs) for the early stage (Table 1).

**Table 1:** Results for the detection of the Mask R-CNN model on the validation dataset without considering the segmentation

Stage	True positive	False negative	False positive	Precision	Recall	F-score
Early	65	3	1	0.98	0.96	0.97
Mid	146	5	7	0.95	0.97	0.96
Late	294	10	26	0.92	0.97	0.94

We also assessed the adequacy of the segmentation for each true positive detection. For this task, we evaluated the IoU between the predicted masks and the ground truth mask. We obtained a mean IoU of 0.79 ± 0.0731 standard deviation (STD) for the early stages, 0.8795 ± 0.0968 for the mid stages, and 0.8631 ± 0.1466 for the late stages (Table 2).

**Table 2:** Statistical descriptors for the segmentation

	Early	Mid	Late
Total	65	146	294
Mean	0.79	0.88	0.86
STD	0.07	0.10	0.15
Min	0.59	0.41	0.20
25%	0.74	0.87	0.87
50%	0.80	0.92	0.91
75%	0.84	0.93	0.93
Max	0.91	0.96	0.97

The value represents the IoU between the model predicted masks and the ground truth masks on correct detections for the validation dataset separated by early, mid, and late stages of development

### Leaf tracking

**Fig. 2:**
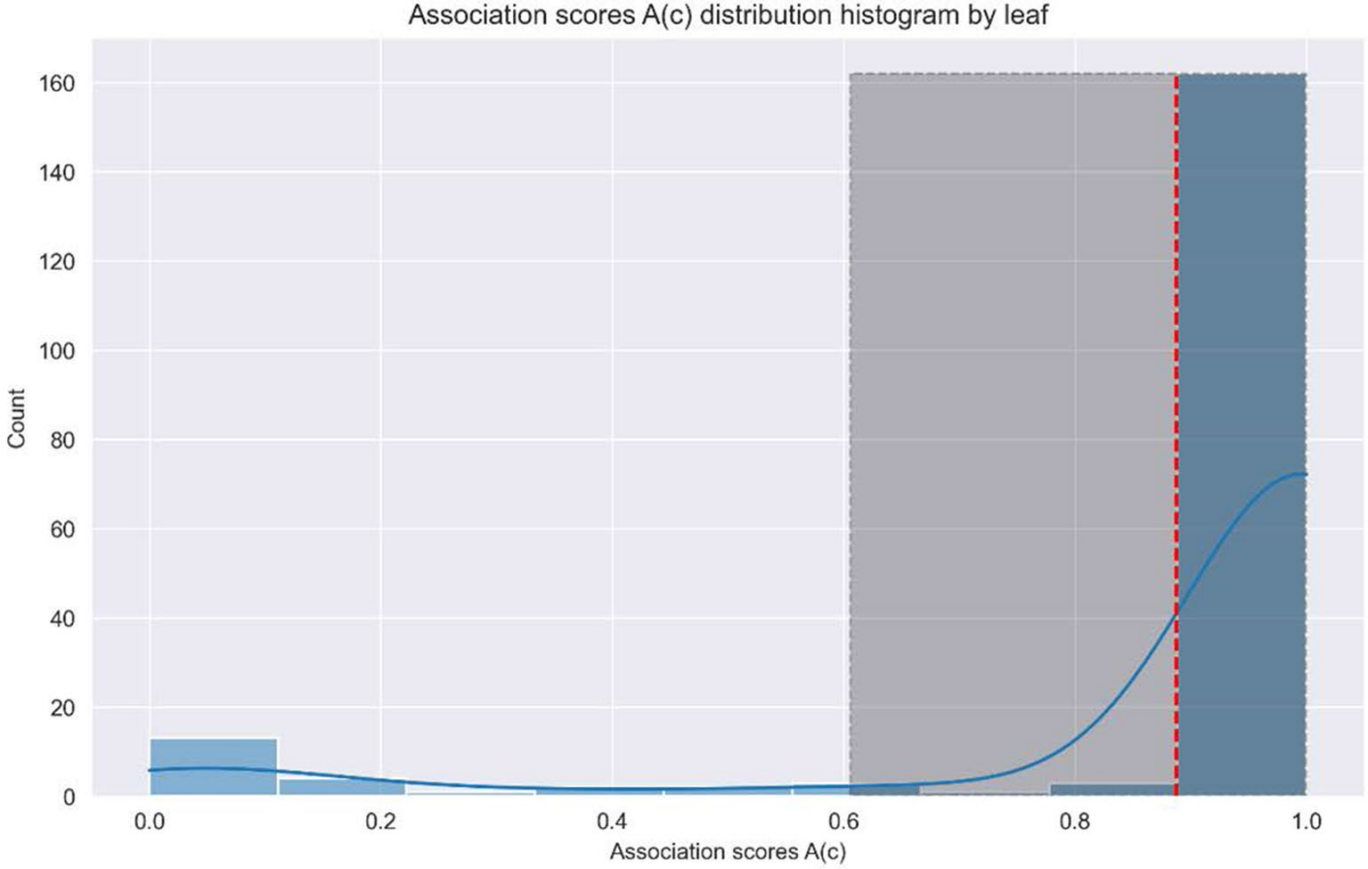
Distribution of the Association metric A(c) score counts of the HOTA metric. The red line indicates the mean A(c) score. The blue line indicates the density, and the grey box outlines the STD range of the A(c) score

The HOTA metric was evaluated per individual, which achieved a mean score of 0.84603 ± 0.0623 for nine plants evaluated through 57 timepoints each. The lowest HOTA achieved was 0.7452 and the highest 0.9879. A total of 162 out of 191 leaves were tracked from beginning to end without major issues detected. This accounts for 84.8% of the tracking evaluation dataset (Fig. 2).

### Leaf count as indicator of development progression

**Fig. 3:**
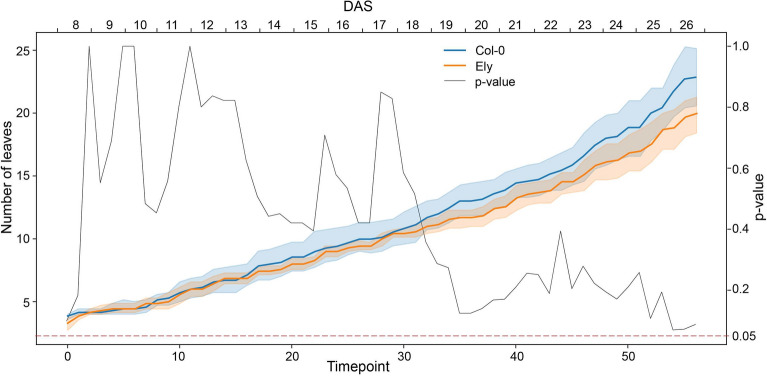
Rosette leaf number of *A. thaliana* accessions Col-0 (blue) and Ely (orange) determined over time, either plotted against timepoint or days after sowing, DAS. The minimum and maximum numbers of leaves are shown as shaded regions around the mean of seven replicates (solid line) for each accession. A t-test was performed for every time point to determine the p-values for statistical significance of the difference in leaf number between these two accessions over time (gray solid line)

The total number of rosette leaves over time was retrieved for Col-0 and Ely to evaluate the developmental rate during vegetative growth over the course of the experiment until 26 DAS (Fig. 3). In the first recorded images on 8 DAS, plants generally had two or three true leaves and two cotyledons. There was no difference between Col-0 and Ely in the leaf count in early days, but a difference became apparent when plants grew older. At the last timepoints, Col-0 had four leaves more than Ely (p-value close to 0.05, Fig. 3), which indicates a slightly higher growth rate than Ely. At the time the experiment was finished, Col-0 and Ely were not yet flowering or bolting, which means that the reported leaf numbers are not the final total leaf numbers that these genotypes could make during their vegetative phase.

### Photosynthetic capacity at plant level

**Fig. 4:**
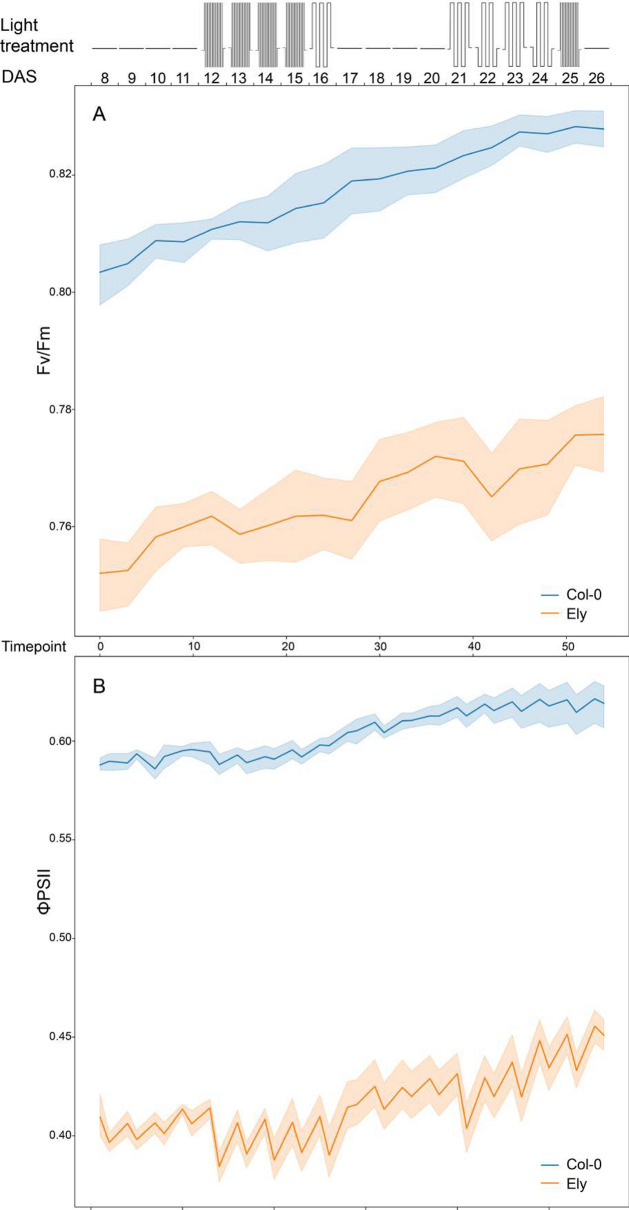
Photosynthesis dynamics of *A. thaliana* accessions Col-0 (blue) and Ely (orange) at individual plant level. Schematic representation of the fluctuating light treatment over time is shown in the top panel, with 300 µmol m^−2^ s^−1^ of constant irradiance, 100 µmol m^−2^ s^−1^ of low irradiance and 900 µmol m^−2^ s^−1^ of high irradiance levels. Maximum quantum yield (F_v_/F_m_, **A**) and photosystem II efficiency (ϕPSII, **B**) are plotted against time (as timepoint or as days after sowing, DAS). The variation for these traits is shown as shaded regions around the mean of 8 replicates (solid line) for each accession

The photosynthetic capacity at individual plant and leaf level was evaluated as maximum quantum yield (*F*_*v*_/*F*_*m*_) and as operating efficiency of photosystem II (ϕPSII). At plant level, Col-0 had a significant better photosynthesis capacity than Ely, with *F*_*v*_/*F*_*m*_ often 0.04–0.05 higher (roughly 5%, Fig. 4A) and ϕPSII between 0.18–0.22 higher (roughly 50%, Fig. 4B). Both *F*_*v*_/*F*_*m*_ and ϕPSII of Col-0 and Ely increased over time, while plants grew. The fluctuating light treatments affected both photosynthetic capacity parameters of Ely but not of Col-0. *F*_*v*_/*F*_*m*_ of Ely increased gradually in days with constant light, but remained more or less constant during fluctuating light treatments (Fig. 4A).

The diurnal response of ϕPSII in Ely was emphasized during days with fluctuating light treatments, compared to constant light days, which was not or much less obvious for Col-0 (Fig. 4B).

### Photosynthetic capacity at leaf level

**Fig. 5:**
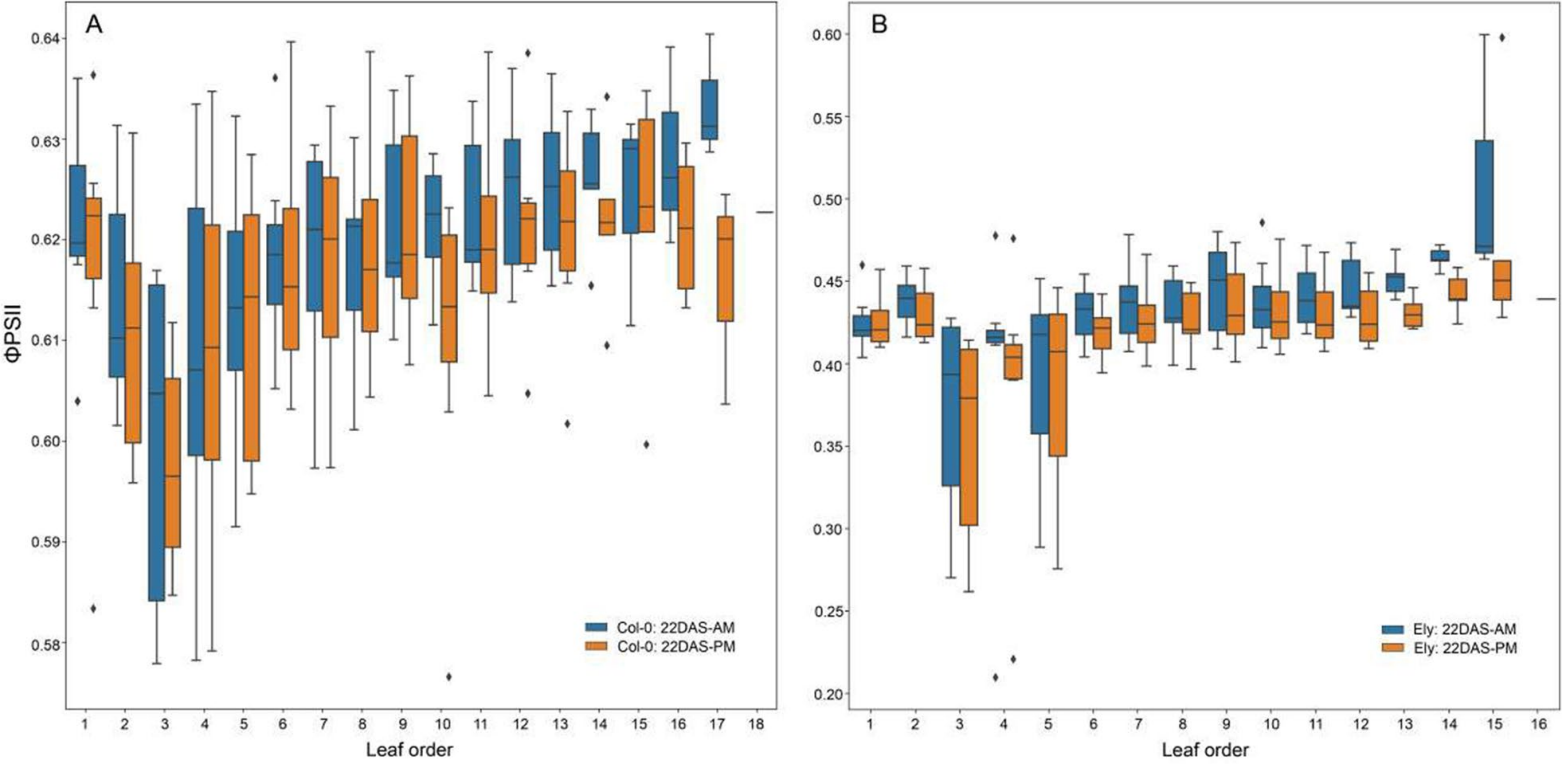
Photosystem II efficiencies (ϕPSII) of individual leaves of *A. thaliana* accessions Col-0 (**A**) and Ely (**B**) before and after a fluctuating light treatment. Boxplots represent the ϕPSII measurements of individual leaves of 8 replicate plants at day 22 after sowing (22 DAS) in both morning before the fluctuating light treatment (AM in cyan) and in the afternoon (PM in orange). Leaf order (x-axis) was obtained according to the order of emergence and detection over the time, meaning the higher leaf order the younger leaf and vice versa

**Fig. 6:**
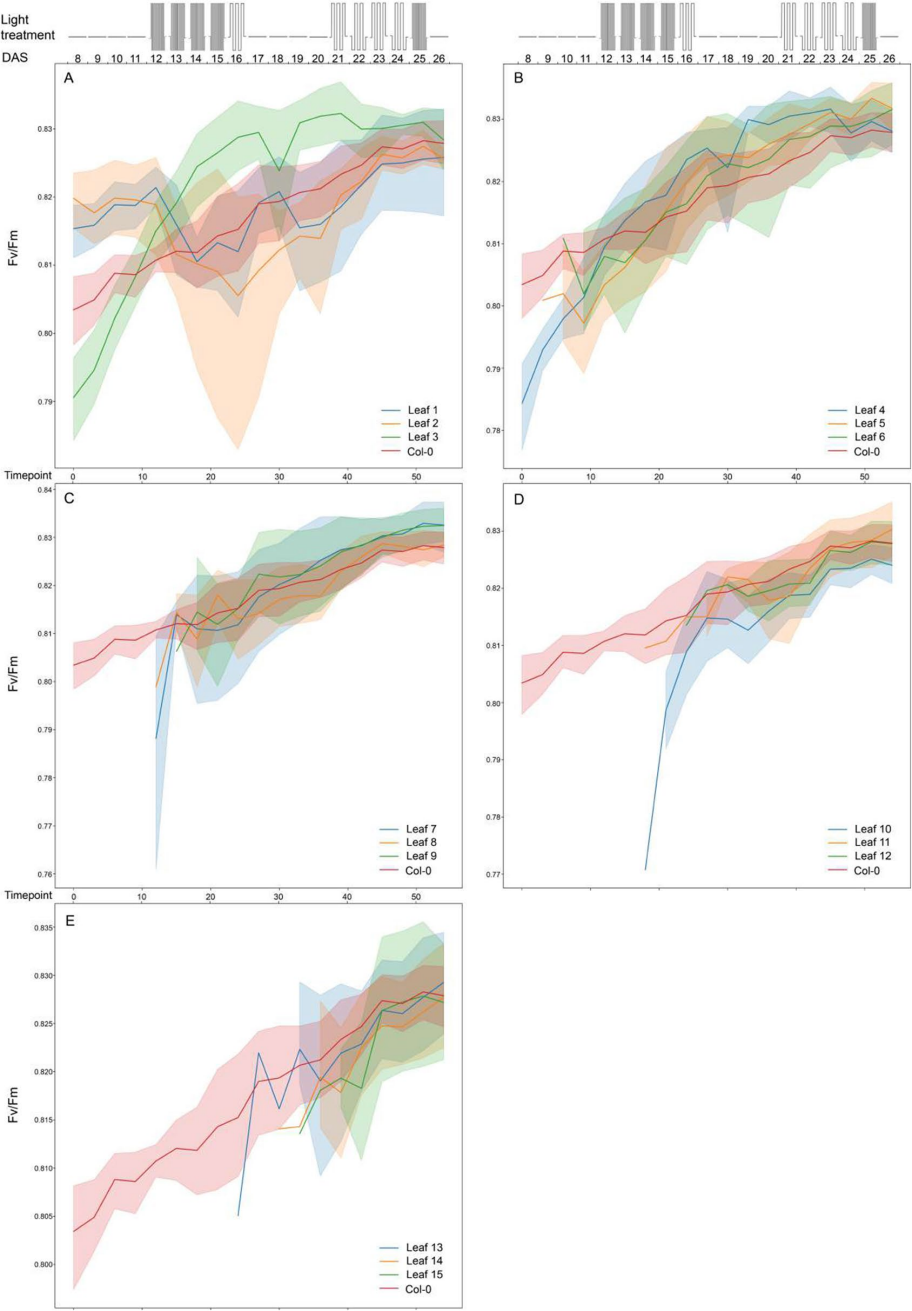
Maximum quantum yield (*F*_*v*_/*F*_*m*_) of whole Col-0 rosettes and 3 indicated individual leaves per panel (leaf number indicated: (**A**) for leaf 1–3, (**B**) for leaf 4–6, (**C**) for leaf 7–9, (**D**) for leaf 10–12 and (**E**) for leaf 13–15) determined over time (as timepoint or as days after 18 sowing, DAS). Schematic representation of the fluctuating light treatment over time is shown in the top panel, with 300 µmol m^−2^s^−1^ of constant irradiance, 100 µmol m^−2^s^−1^ of low irradiance and 900 µmol m^−2^s^−1^ of high irradiance levels. The mean F_v_/F_m_ values are shown with solid lines, with shaded regions above and below indicating standard errors of the means. Data points represent 8 replicates

**Fig. 7:**
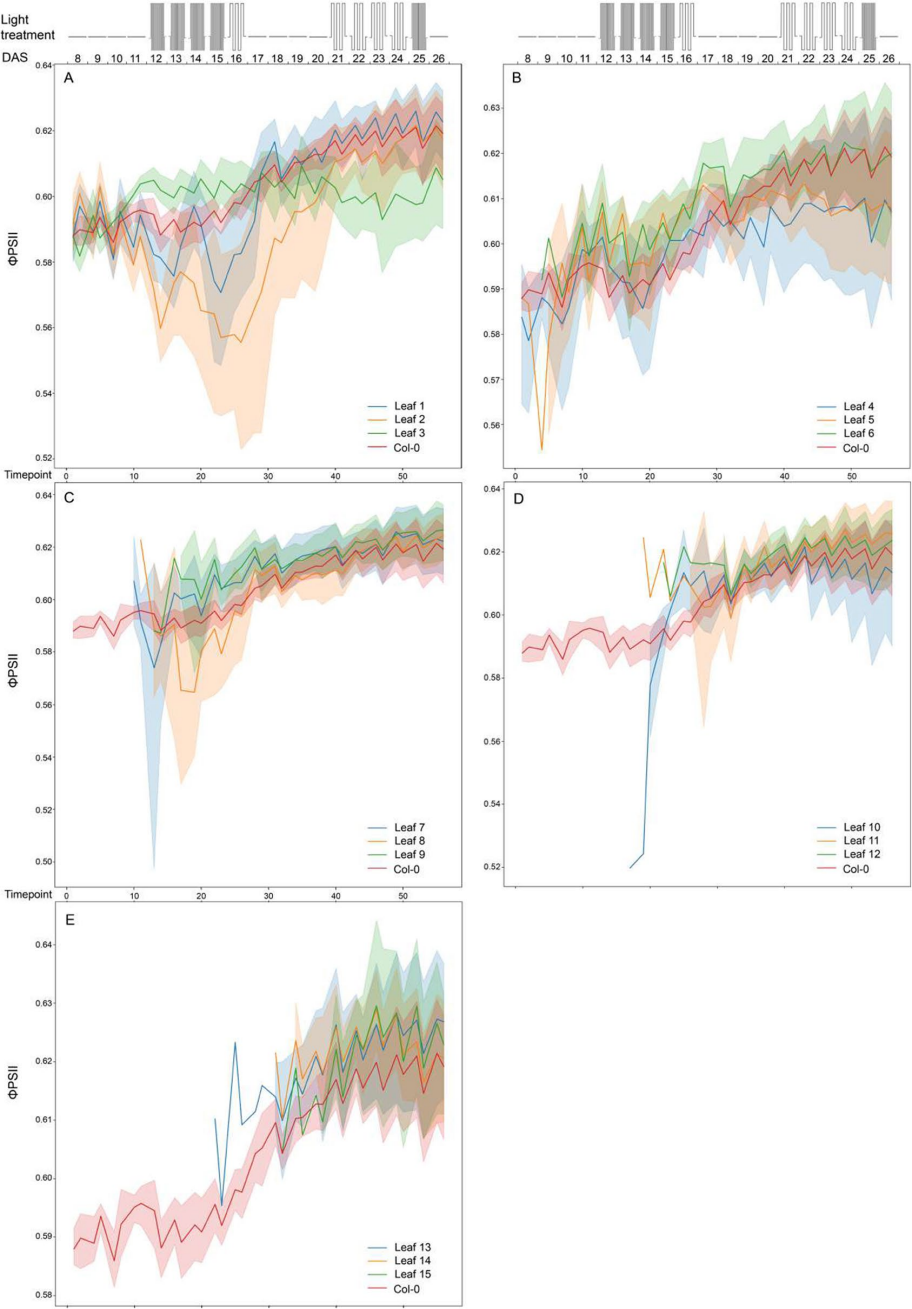
Photosystem II efficiency (ϕPSI) of whole Col-0 rosettes and 3 indicated individual leaves per panel (leaf number indicated: (**A**) for leaf 1–3, (**B**) for leaf 4–6, (**C**) for leaf 7–9, (**D**) for leaf 10–12 and (**E**) for leaf 13–15) determined over time (as timepoint or as days after sowing, DAS). Schematic representation of the fluctuating light treatment over time is shown in the top panel, with 300 μmol m^−2^s^−1^ of constant irradiance, 100 μmol m^−2^ s^−1^ of low irradiance and 900 μmol m^−2^s^−1^ of high irradiance levels. The mean Fv /Fm values are shown with solid lines, with shaded regions above and below indicating standard errors of the means. Data points represent 8 replicates

**Fig. 8:**
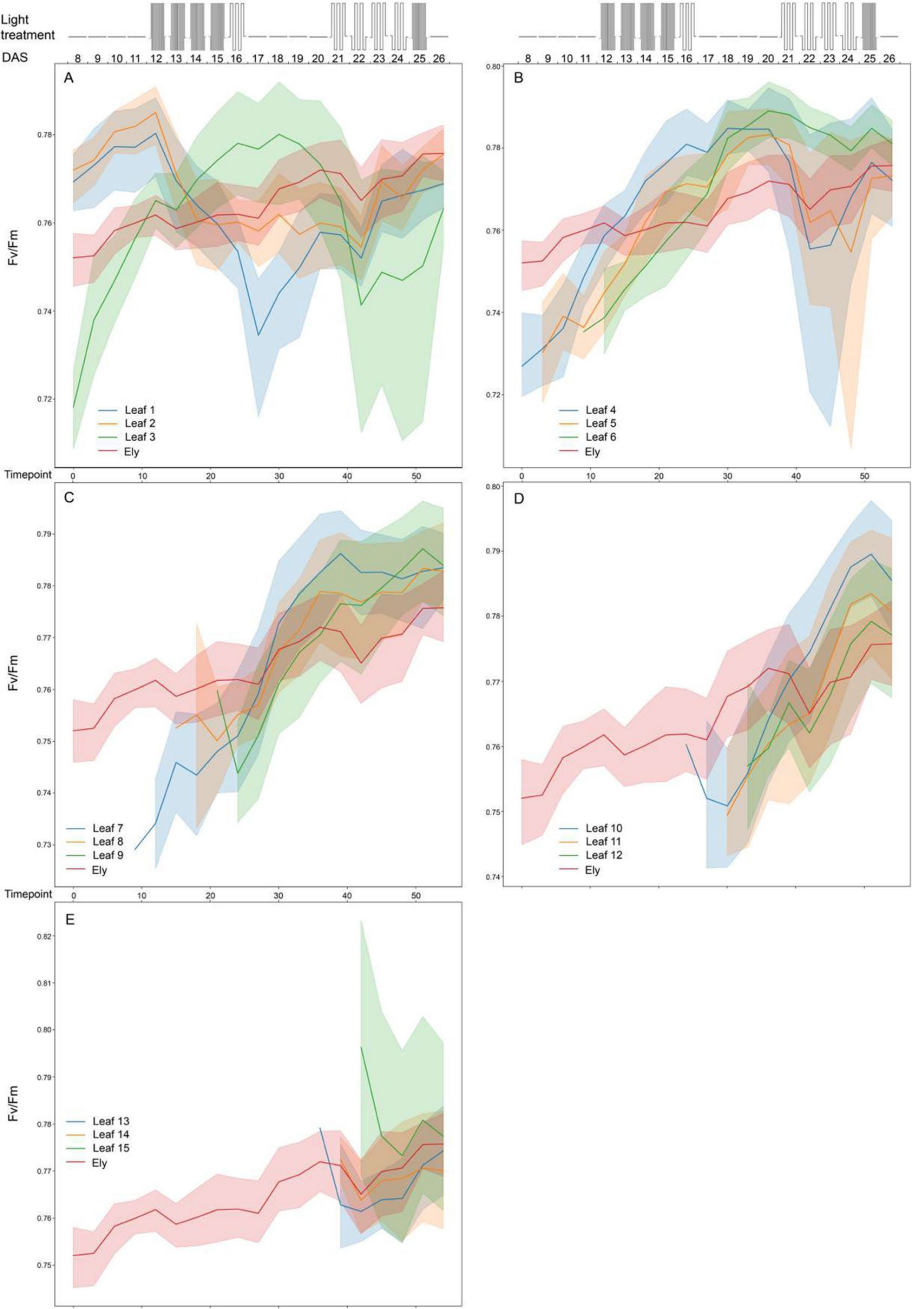
Maximum quantum yield (Fv /Fm) of whole Ely rosettes and 3 indicated individual leaves per panel (leaf number indicated: (**A**) for leaf 1 to 3, (**B**) for leaf 4 to 6, (**C**) for leaf 7 to 9, (**D**) for leaf 10 to 12 and (**E**) for leaf 13 to 15) determined over time (as timepoint or as days after sowing, DAS). Schematic representation of the fluctuating light treatment over time is shown in the top panel, with 300 μmol m^−2^ s^−1^ of constant irradiance, 100 μmol m^−2^ s^−1^ of low irradiance and 900 μmol m^−2^s^−1^ of high irradiance levels. The mean Fv /Fm values are shown with solid lines, with shaded regions above and below indicating standard errors of the means. Data points represent 8 replicates

**Fig. 9:**
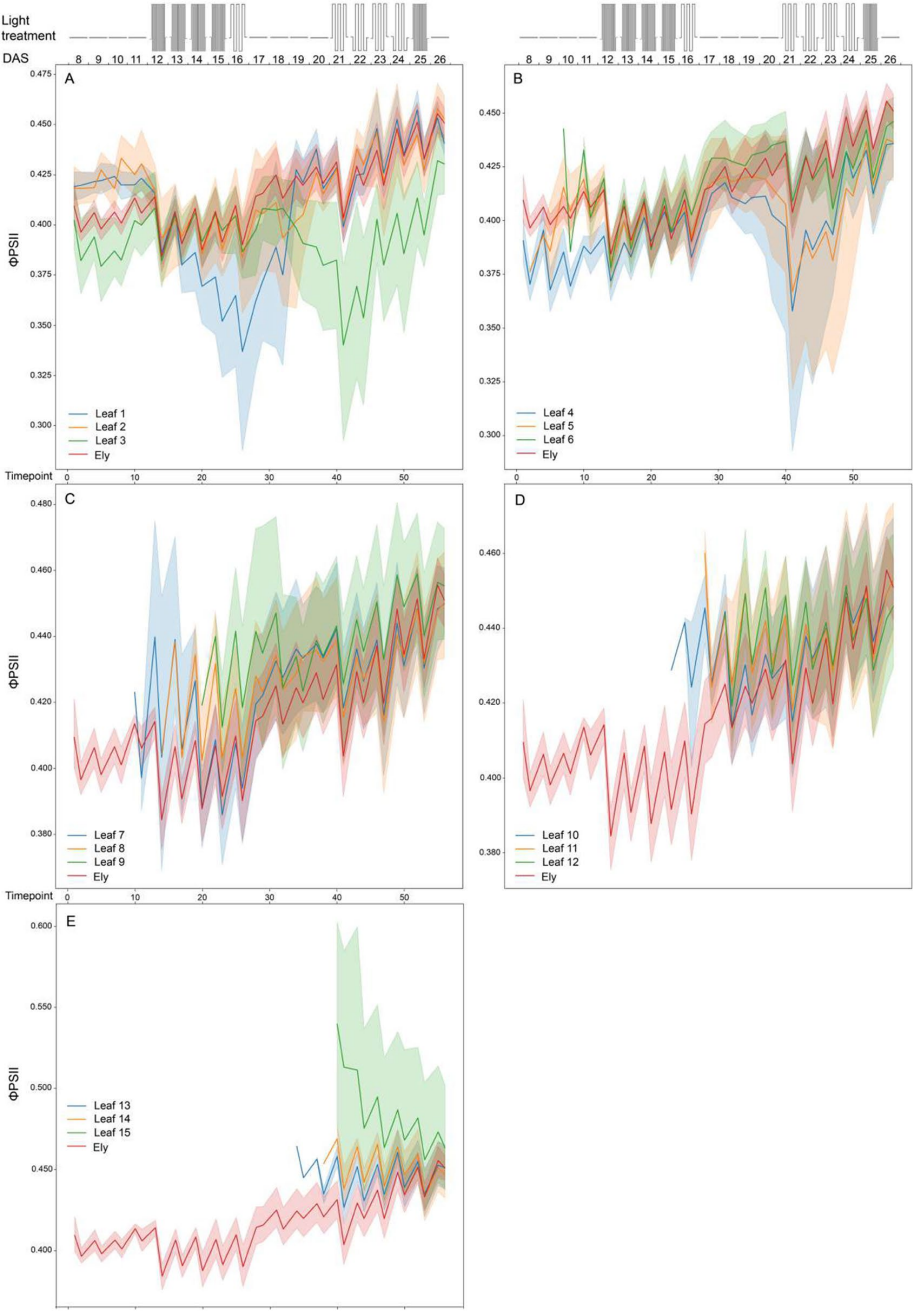
Photosystem II efficiency (ϕPSII) of whole Ely rosettes and 3 indicated individual leaves per panel (leaf number indicated: (**A**) for leaf 1–3, (**B**) for leaf 4–6, (**C**) for leaf 7–9, (**D**) for leaf 10–12 and (**E**) for leaf 13–15) determined over time (as timepoint or as days after sowing, DAS). Schematic representation of the fluctuating light treatment over time is shown in the top panel, with 300 μmol m^−2^ s^−1^ of constant irradiance, 100 μmol m^−2^ s^−1^ of low irradiance and 900 μmol m^−2^ s^−1^ of high irradiance levels. The mean Fv/Fm values are shown with solid lines, with shaded regions above and below indicating standard errors of the means. Data points represent 8 replicates

*A. thaliana* plant photosynthesis is expected to change over time depending on plant development. During the vegetative stage, with plants still in the rosette stage, this will be mainly affected by leaf age and leaf development [8]. Upon identification of individual leaves, the dynamics of leaf photosynthetic parameters *F*_*v*_/*F*_*m*_ and ϕPSII over time can be determined, as shown for both Col-0 and Ely in Figs. 5, 6, 7, 8 and 9. Leaf photosynthesis generally follows the same trend as observed for whole plant photosynthesis, with a degree of amplitude difference. Leaves 1, 2 and 3 are exceptions (Figs. 5, 6A, 7A, 8A, 9A), likely because they are not properly identified, and represent a mixture of cotyledons and true leaves between replicates. The reason is that in the first input picture, there were four to five leaves detected and randomly assigned. Unexpectedly, *F*_*v*_/*F*_*m*_ of true leaves had a different pattern than individual plant mean in the Ely accession (Fig. 8). While *F*_*v*_/*F*_*m*_ of Col-0 true leaves had a relatively similar pattern compared to that of individual plant mean (Fig. 6), Ely leaf *F*_*v*_/*F*_*m*_ kinetic showed that *F*_*v*_/*F*_*m*_ of a leaf increased sharply (roughly from 0.72 to 0.79), reached its maximum potential when leaves were around a week old and then decreased. As a result, there is substantial variation for *F*_*v*_/*F*_*m*_ between leaves in Ely at any given moment of time.

### Photosynthetic response is influenced by leaf age and order

Leaf tracking also allows evaluation of the influence of leaf age/order on ϕPSII over time and in response to fluctuating light treatments. Figure 5 shows the leaf ϕPSII measured in the morning and afternoon at 22 DAS for Col-0 and Ely. Between these measurements, plants were exposed to fluctuating irradiance between 100 and 900 µmol m^−2^ s^−1^ for 5 h, with duration of each irradiance level taking 60 min. The ϕPSII response is determined as the change in ϕPSII before and after the fluctuating irradiance treatment. At individual plant level, the ϕPSII response was small, 0.0025 for Col-0 and 0.01 for Ely (Fig. 4B). However, at the leaf level, younger leaves showed a much larger ϕPSII response than did older leaves (Fig. 5). The ϕPSII response of each individual plant was therefore mainly determined by the response of the younger leaves.

## Discussion

The aim of this work is to provide both a pipeline and an implementation to perform leaf tracking on top-view images of plants in time series. In this case, the model has been trained in a model organism well known by the plant science community, *Arabidopsis thaliana*, however this pipeline can be applied to any other plant species as long as they can be properly described in a top-view image. Leaf detection and segmentation, by itself, is a task that has been performed through different approaches and in different contexts and challenges [13]. It is clear from the multitude of approaches that this problem can be tackled in different ways. E.g. edge classification has been used to improve leaf segmentation [18], but also a combined statistical graph-based method has been used [19] Recent advances in the computer vision field allowed us to implement a novel, more direct approach using convolutional neural networks. The use of mask R-CNN is extensive, and it achieves great results in multiple tasks, with different kinds of objects [20]. Numerous technical alternatives exist for detection and segmentation; however, these alternatives typically specialize in either instance detection or segmentation, but not both. Mask R-CNN stands out as it is well-documented and can be easily fine-tuned using PyTorch, enhancing its accessibility to the community. Thus, it was a suitable choice for our case. In this case, the model achieves the best results for the detection of early leaves but scores lower for segmentation at that stage. In contrast, the lower results for detection are on the last stages but then it achieves good results on the segmentation. This might be due to the size of the image, with more difficulty to segment a low-resolution object with small size but less difficulty to detect it as the background contains fewer interfering objects, such as other leaves. On the other hand, it might be more challenging to detect individual objects in a crowded image but easier to segment when the detection is right. One issue we found regarding the detection of larger leaves is a relatively high rate of false positives (26 FP out of 330 detections, at a precision of 0.91). This could be addressed by adding an adaptative confidence threshold depending on the stage of the plant, to better balance the trade-off between precision and recall, or by increasing the training dataset size. Recent work has proposed the tracking of dynamic changes at leaf-specific resolution in *A. thaliana* [21] but the proper individualization of different leaves, without recurring to neural networks, has proven to remain a challenge. Through this method, the real challenge lies more on the tracking side than on the individualization of the different leaves. On the other hand, older work provided a method for tracking [15], but the implementation can be difficult for a standard user with medium coding experience. A concept such as the overlap between figures (IoU) can be easily implemented in conjunction with the masks produced by a model given on the PyTorch framework and results in a very intuitive pipeline for most of the users.

The approach of using a mixture between AI and distance measurement, as shown here, has proven to be efficient in other cases as well, such as cell and particle tracking [22]. While there are several technical alternatives to the IoU metric for tracking, such as optical flow or center proximity, our implementation has demonstrated sufficient accuracy for leaf tracking, as evidenced by its high accuracy and HOTA scores. Regarding the limitations of the implementation, most of the issues in association arise from false negatives in the detection process. As mentioned in the methodology, a big enough gap between a mask at time frame n and n + 1 may produce an association failure. Consequently, failing to keep track for more than 3–4 frames of time allows for enough leaf growth to detriment the method. On the other hand, improving the detection and segmentation accuracy may result in a better association, which can be easily achieved by expanding the number of examples on the training dataset. The biggest challenge to solve is the effect of leaf movement during day-night transition. In certain *A. thaliana* accessions, the leaf movement of the first true leaves can be so strong that there is no overlap between time frames, which is likely to result in a total loss of association and a new mask assignation. This issue may be tackled by adding one or two images in the transition period from day to night and vice versa, helping to soften the abrupt changes in position. High precision in detection is crucial for accurate leaf tracking, as consecutive detection failures of the same leaf may result in lost tracking due to plant growth. Acknowledging this, we chose the HOTA evaluation metric, which penalizes both failures in mask association and errors related to mask confusion and missed detections. This metric enables us to effectively evaluate the method's quality and understand its robustness.

On the possible usages and utility, this method allows for individualized leaf growth tracking, and also allows for projected leaf surface area measurement and, depending on the equipment of the phenotyping platform, in fluorescence and infrared measurements per leaf. This kind of analysis may provide insight on differences between individual plants’ leaves or patterns within rosettes, providing new traits for genetic association studies. This method is also relatively inexpensive in terms of data to implement, as it is based on fine-tuning instead of full training of the detection model. Removing the need for a full training translates into less labelling and less time spent in preparing a dataset, reducing the data preparation burden that normally comes with the use of convolutional neural networks, and also the computational costs. As for usage recommendations, life sciences researchers who wish to utilize our implementation in their own workflows should note that the provided output includes raw data containing masks that may have lost tracking as well as false positives. Therefore, prior to conducting data analysis, it is recommended to filter out such data to minimize the introduction of background noise. The detection and segmentation model has been trained on FC images produced by the phenotyping hardware, which makes this software ideal for phenotyping platforms using the same or comparable hardware. If working with images from a different origin (i.e., RGB images or infrared), the model should be fine-tuned to obtain the best results. This can be easily achieved by labeling a small dataset and running the scripts provided in the code repository. In case of applying the model to a different plant species, the mask R-CNN model should be retrained on examples of that species. It is important to consider that this methodology works on plants that can be described as top-down images, meaning that plants with high 3D growth will need a different methodology due to the high level of overlap between leaves.

Last, in this manuscript we aim to encourage further development of new methods for better individualization of leaves per plants, therefore the train, test and validation datasets used to train the model are provided, allowing users to expand the library with different approaches for either the segmentation or the tracking. As a case study, we used the CF images to determine photosynthetic capacity at individual plant and leaf level in the *A. thaliana* accessions Col-0 and Ely over time and under various light treatments. The photosynthetic capacity of Ely is lower than that of Col-0 at individual plant level (Fig. 4), which is consistent with previous studies [23, 24], which explained this to be due to a mutation in the chloroplast *PsbA* gene, encoding the D1 protein of photosystem II, of Ely. The response to light treatment was also more pronounced in Ely than in Col-0. Our results show that younger leaves have a higher photosynthetic capacity than older leaves (Fig. 5), which is likely to be due to a higher ability to redesign leaf anatomy and the photosynthesis machinery for acclimation to a fluctuating light environment [8]. This is the first time that leaf photosynthesis dynamics is evaluated in a nondestructive and automated manner. Variation for photosynthesis parameters between leaves was observed although the differences are subtle. Nevertheless, these potentially enlarge the variation in photosynthesis parameters observed between genotypes, which is often smaller when determined at individual plant level [6, 7]. Variation in leaf level photosynthesis parameters is of great interest for quantitative genetic analysis and may resolve the effect of developmental differences on photosynthesis in genetically segregating populations with variation in plant growth among genotypes.

## Conclusion

In this work a method for individual leaf segmentation and tracking on top-view images is provided, as well as an example of implementation of the method. Although there are many technical alternatives, we find this method to be efficient and easy to use for automated workflows on phenotyping platforms such as the one we used. The advantages provided by the use of convolutional neural networks and recent models are clear on precision at detection and individualization, limiting the challenges to tracking. Using a simple concept such as masks overlap, tracking can be solved to a certain degree of confidence, which can be further improved through expanding the training dataset for leaf detection. The basic requirements in data for fine-tuning the detection model are low, giving users the opportunity to take advantage of pretrained weights with little cost.

## Methods

### Plant material and growing conditions

A set of 177 *A. thaliana* accessions was used, which includes 175 accessions of the regional Dutch population [25], and the Ely and Columbia-0 (Col-0) accessions. Seeds of accessions were first pregerminated on filter paper with demi water in petri dishes by stratification treatment for five days, after that they were germinated at 24 °C for 16 h. The germinated seeds were transferred to rockwool block (4 × 4 cm; GrodanTM) to germinate. From this point onwards, the days of the experiment were referred as days after sowing (DAS). This set was grown in a complete randomized block design, with eight blocks. There was a replicate in each block for every Dutch accession, and four replicates were available for Col-0 and Ely, in total 1440 individuals.

Plants were supplied with standard Hyponex nutrient solution [6]. The climate room was set to 20 °C and 18 °C, day and night temperature, respectively. Photoperiod was set to 12 h (light on from 8 am to 8 pm), in which light intensity increased and decreased gradually in the first and last hour of the photoperiod to resemble dawn and dusk of the day. Irradiance stayed at 300 µmol m^−2^ s^−1^ until application of light treatment for five hours (from 11 am to 4 pm), then returned to 300 µmol m^−2^ s^−1^. There were three light treatments: constant irradiance at 300 µmol m^−2^ s^−1^, fast fluctuation and slow fluctuation between 900 and 100 µmol m^−2^ s^−1^. The duration of each irradiance level taking 15 and 60 min, respectively, in the fast and slow light fluctuation treatment. photosynthesis, CF measurements were taken once per day in the night.

### Phenotyping and data collection

Photosynthetic parameters were measured based on chlorophyll fluorescence (CF) imaging every day from 8 DAS for 19 days with the Phenovator (PlantScreen Robotics XY system, Photon System Instruments*™*). Re-emitted light from photosystem II (PSII) in form of fluorescence is measured and compared whilst exposing plant to a combination of actinic lights (lights that drives photochemistry and photosynthesis), darkness and series of saturating pulses. This method is known as pulse-amplitude modulated fluorescence analysis, and provides non-invasive assessment of PSII efficiency to pass electrons to photosynthetic apparatus [26, 27]

In dark-adapted state, a minimum value of CF (*F*_0_) is measured by very low measuring light, which can only result in minimal level of emitted CF but not electron transport induction. Application of saturating pulses in dark-adapted state, driving closure of all reaction centers, induces maximum level of CF (*F*_*m*_). The difference between Fm and F0 is variable fluorescence, termed *F*_*v*_, from which *F*_*v*_/*F*_*m*_ parameter can be calculated, which is used as robust indicator of maximum quantum yield of PSII photochemistry.

Similarly CF is measured at light-adapted state using actinic light, which results in *F*_*p*_. Saturating pulses can be applied during the actinic light exposure, thus *F*_*mp*_ is obtained. The difference between *F*_*mp*_ and *F*_*p*_ in proportion to *F*_*mp*_ is used to determine the operating efficiency of PSII photochemistry (ϕPSII). There were three CF imaging a day, consisting of one for dark-adapted maximum quantum yield of PSII (*F*_*v*_/*F*_*m*_) and two for light-adapted PSII efficiency at 9.45 am and 5.15 pm. The dark-adapted measurement was performed in the first hour of the day (12 am), thus in total 19 measurements are available. The light-adapted measurements, were made in the morning (9.15 am) and afternoon (5.15 pm) during the photoperiod before and after the light treatment, which led to 38 measurements. Thus for the whole course of the experiment, there were a total of 57 CF measurements. Data collected from the CF imaging was used to produce grayscale images by normalization in a range from 0 to 1 of the data matrices.

### Training and validation datasets for detection and segmentation, and tracking evaluation dataset

For the training, we used an integrated dataset of *A. thaliana* plants at different stages of development ranging from the display of the first true leaves to the largest size on day 26 after sowing. This dataset was composed of 166 individual plant images with a total of 1773 leaves, from which 149 images were used as subset for training and 17 for testing. A different dataset, composed of 18 individuals in three different developmental stages, was used for validation. The validation dataset was grouped in three categories, early stage (17 images, one removed for not displaying any leaves yet), mid stage (18 images) and late stage (18 images), adding to a total of 53 images (523 leaves) of the same 18 individuals.

To estimate the efficiency of the tracking we used a separate dataset of 9 plants through 57 time points, with a total of 204 leaves. This dataset was never used, neither in the training–testing nor in the validation of the Mask R-CNN model for leaf detection and segmentation.

### Detection and segmentation

Detection and segmentation have been performed through a convolutional neural network-based approach by selecting a mask R-CNN model [14] which can tackle the objectives of detection and segmentation in an efficient way. For the feature extraction, we employed a resnet50 backend [28] and fine-tuned the full model parting from pre-trained weights on the MSCOCO dataset [29] with a number of classes for classification set to two (leaf and background). The training was performed using stochastic gradient descent (SGD), with a learning rate 0.005 over ten epochs. The original dataset and more details on the training can be found in the code repository as well as notebooks to replicate it. To evaluate the model, we scored the validation dataset in two different contrasts. The initial comparison focused on precision and recall for leaf detection, assessing the model ability to accurately detect leaves while minimizing false positives. We discarded detections with a confidence below 0.9 and counted the number of correct detections against the total number of false positives and false negatives. A correct detection was only assigned if the IoU between the model prediction mask and the ground truth mask was at least of 0.15. If a predicted mask was found below this threshold and no ground truth mask could be assigned, then it was detected as false positive. Conversely, if a ground truth mask was missing a suitable predicted mask, it was labelled as a false negative.

The second contrast was the measurement of the IoU (Intersection over union) between the ground truth and the predicted masks. Based on the same criterion, we found the pairing mask by discarding detections below the 0.9 confidence and by getting the best overlapping mask for each leaf. In this test, false positives and negatives are disregarded as the aim is to measure the segmentation performance in case of a true positive. Both contrasts were applied and evaluated separately on each of the categories defined in the validation dataset and their respective metrics were collected for each of them.

### Instance tracking

**Fig. 10:**
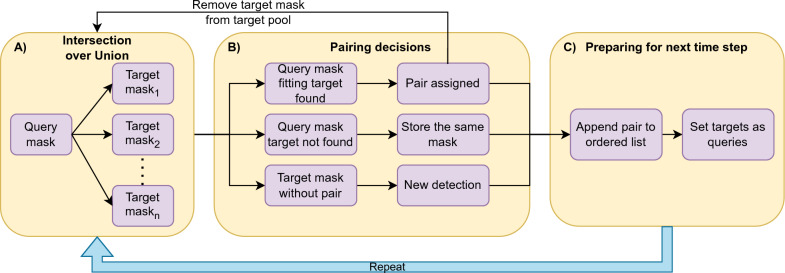
Flowchart illustrating the tracking process. Panel **A** depicts the initial step, where the mask for time step n (query) is retrieved and the IoU is computed for all masks at time step n + 1 (targets). In Panel **B**, the decision loop is made based on the resulting IoU. If the query and target match, the pair is assigned, and the target is removed from the target pool. If the query is not found, indicating that the leaf is either covered in the image or the detection failed, the position is stored by saving the same mask. Finally, the remaining target masks are considered as new detections. Panel C shows the list of masks, in order, which is stored and set as the new queries for the next time step

The approach used for leaf tracking was based on overlapping masks between timepoints (Fig. 10). As most of the leaf changes in *A. thaliana* development can be traced on two dimensions, masks between timepoint n and n + 1 will be largely overlapping if the timestep or the variance between instances are small enough. In this case the largest time frame is roughly 8 h, which represents a small enough gap between different developmental stages of the plant. At each time point, the IoU between a query mask and all the target masks in the next time point was estimated. The target mask with the highest IoU and above a certain threshold (0.15 in this study) was assigned as the respective pair. We then generated a dictionary containing the query-target relation and appended it to a list, which will keep the order. Thereafter, the target was removed from the target pool. If the query was not found within the targets, we created a pair dictionary with itself and appended it to the list. After looping over all the queries, the remaining targets were assumed to be new leaves, which were appended to the same list as dictionaries containing empty queries and the mask as a target. Once all the pairs had been assigned, we collected all the targets from the list preserving the order and created a new query dataset for the next time step. This process was repeated until we reached the last time step (Fig. 10).

To evaluate the quality of the tracking we used the HOTA metric [30] and estimated the total leaves that were tracked from the first to the last time point (Last frame it could be detected) without missing the association of the masks and with a maximum of five missing detections for that leaf. This way we generated two categories, right and miss. Thirteen leaves from the tracking evaluation dataset were excluded from both metrics due to limited time points (1–3 time points) or small detection errors (very low area), resulting in a total of 191 leaves considered for the evaluation of the metrics.

### Leaf counting and photosynthesis capacity analysis of two different genotypes

The two accessions Col-0 and Ely were selected as genotypes for a comparative experiment. For each genotype, seven replicates were considered. To retrieve the total number of rosette leaves per time point the ϕPSII file produced by the system was used. A summary of the number of instances tracked at each time point was sufficient to perform the estimation. With the leaf number per replicate obtained, a t-test was performed at each time point to evaluate the significance of the differences between genotypes. The estimation of *F*_*v*_/*F*_*m*_ and ϕPSII per individual plant and leaf was performed on the raw data based on pixel means. Instance segmentation masks were applied to the raw matrix (fimg produced by the phenotyping system) to extract the raw values for the region and mean values were estimated per instance.

### Metrics definition

Precision is defined as the ratio between true positives detected against the total detections retrieved by the model.

1 $$Precision = \frac{True\;Positives}{{True\;Positives\;(TP) + False\;Positives(FP)}}.$$

Recall measures the sensitivity of a model taking in consideration True positives against the ground truth detections.

2 $$Recall = \frac{True\;Positives}{{True\;Positives\;(TP) + False\;Negative\,(FN)}}.$$

F-score is the harmonic mean of precision and recall.

3 $$F{\text{-}}score = 2*\frac{precision*recall}{{precision + recall}}.$$

Intersection over Union of two figures (A and B) is defined as the symmetric difference of figures A and B against the union of those figures.

4 $$Intersection\;over\;union\;(IoU) = \frac{|A \cap B|}{{|A \cup B|}}.$$

The Higher Order Tracking Accuracy (HOTA) metric is defined as in the published document [30]. This measure joins detection and association scores into a single value.

5 $$Higher\;Order\;Tracking\;Accuracy\;(HOTA) = \sqrt {\frac{{\sum\nolimits_{{c \in \{ TP\} }} {A(c)} }}{|TP| + |FN| + |FP|}.}$$

6 $$A(c) = \frac{|TPA(c)|}{{|TPA(c)| + |FNA(c)||FPA(c)|}}.$$

## Data Availability

All the code in this project was developed using python and different public libraries. The main libraries used in this project were PyTorch [31] for the neural networks, PIL and OpenCV [32] for the image manipulation, Pandas [33] and NumPy [34] for data analysis, and scikit-learn [35] for the IoU. Part of the PyTorch-Vision GitHub repository was used for the model training, specifically the reference scripts found in the folder detection. These scripts are included in the project GitHub under the modelTraining folder without relevant modifications. The full library of this project is available as a public repository at GitHub https://github.com/Fedjurrui/Leaf-Tracking.

## Abbreviations

HTTP: High-throughput phenotyping.
HOTA: Higher order tracking accuracy
CF: Chlorophyll fluorescence
ϕPSII: Efficiency of photosystem II
PSI: Photon systems instruments
NPEC: Netherlands Plant Eco-phenotyping Center
CNN: Convolutional neural networks
IoU: Intersection over union
TP: True positives
FP: False positives
FN: False negatives
*F*_*v*_/*F*_*m*_: Maximum quantum yield
*F*_0_: Minimum value of CF
*F*_*m*_: Maximum level of CF
